# Effect of Degree of Substitution and Polymer Ratio on the Structure of Chitosan: Carboxymethyl Starch (Bio)Polyelectrolyte Complexes

**DOI:** 10.3390/polym16243539

**Published:** 2024-12-19

**Authors:** David Castro, Valentina Brovina, Mikhail Litvinov, Aleksandr Podshivalov

**Affiliations:** Center for Chemical Engineering, ITMO University, Kronverkskiy Prospekt, 49, 197101 Saint-Petersburg, Russia; dcastrovargas@itmo.ru (D.C.); vbrovinaa@gmail.com (V.B.); mikhail.litvinov.1996@mail.ru (M.L.)

**Keywords:** carboxymethyl starch, chitosan, polyelectrolyte complex, degree of substitution

## Abstract

In this work, three carboxymethyl starches (CMS) were obtained by the two-step reaction process of carboxymethylation with different degrees of substitution (0.16, 0.33, and 0.36). From these samples, (bio)polyelectrolyte complexes ((bio)PECs) were obtained with chitosan (Chit) by the mixing of individual solutions of polymers (0.25 wt.%) at different volume ratios. The effect of the biopolymer and ionized groups of *z* ratios, pH, and degree of substitution of CMS in the formation of PEC were evaluated by turbidimetry and dynamic light scattering. The results showed that increasing the amount of CMS samples (ratio of *z*) led to an increase in the efficiency of the formation of (bio)PEC using CMS with a high DS value. Using the turbidimetry method for the chitosan and CMS mixtures, it was observed that the formation of (bio)PEC is divided into four transition zones delimited by pH transition points, and the stoichiometric complexation (*z* = 1) is achieved at a pH that displayed morphological changes “*pH_morph_*”, which is a single point for Chit:CMS 1, and for Chit:CMS 2 and Chit:CMS 3, this is a range of 4.9–6.4 and 4.3–6.4, respectively. Analysis of the structural properties of the structures of (bio)PECs by dynamic light scattering was characterized by monomodal distribution, and the main observed effect was associated with an increase in the value of *D*_avg_ with an increase in the ratio of Chit:CMS.

## 1. Introduction

The development and study of novel supramolecular structures, such as polyelectrolyte complexes (PECs), have notably increased in recent years. The obtention of these complexes is based on electrostatic interactions between individual solutions of polyelectrolytes (polycations and polyanions) forming a dense phase separated from the solvent of *z* [1,2]. There are some types of PECs such as water-soluble PECs, which are homogenous systems with smaller PEC particles, formed by large difference molar mass ionic group; turbid colloidal stable PECS, which are in the transition state to phase separation; and complex coacervates, which are two-phase systems [2,3]. The main factors that influence the formation of PECs are the ratio of charges of polycations and polyanions; media parameters such as the concentration of polyelectrolytes, pH, and salt concentration; and the preparation parameters like the mode of mixing, mixing order, and ratio [4]. About the applications, PECs are used in many fields because of their chemical properties and macromolecular structure. Adhesives; drug and gene delivery; emulsifiers; functionalization as therapeutic, targeting, or imaging agents; and rheology modifiers are some of the most common applications of PECs [5,6]. In addition, some self-assembly structures such as adhesives [7,8], films [9,10], gels [11,12], and microcapsules are produced from PECs [13,14].

Some of the polymers used for preparing materials based on PECs are chitosan, a widely available natural, non-toxic, biodegradable, and abundant polysaccharide obtained from chitin deacetylation (polycation) [15], and cassava carboxymethyl starch (CMS). Cassava starch is an abundant biopolymer prepared from the root of the cassava plant (Manihot esculenta) cultivated in tropical regions. It is the third largest source of carbohydrates among vegetables and one of the most important crops worldwide [16,17]. CMS is an ether derivate of the starch product of the reaction of carboxymethylation between activated starch, by sodium hydroxide, with monochloroacetic acid (MCA). The CMS presents negatively charged functional groups (CH_2_COO^−^), high swelling capacity, solubility at ambient temperature, low internal viscosity, and less retrogradation [18,19,20].

Chitosan is actively used in agriculture, aquaculture, and agronomy as an antimicrobial agent and as a soil fertilizer [21,22]. In addition, chitosan is also utilized in the production of food and nutritional supplements. It is also appreciated in medicine and cosmetology, the textile and paper industries, and in packaging, biotechnology, and many other fields [23,24]. In another hand, carboxymethyl starch is widely used as a thickener in food products, as a binder and emulsifying agent in industrial applications, and also in textile industries as a printing thickener. Nowadays, the most recent advances and applications of the CMS are in the pharmaceutical applications as a drug carrier [19,25].

Interest in PECs began in the 1930s with the discovery that some natural polyelectrolytes could interact in aqueous media to form colloidal complexes known as complex coacervates in work carried out by Bungenberg de Jong H. and Kruyt H. [26]. In the 1960s, insoluble PECs were identified using synthetic polymers, and the swelling and plasticizing properties of aqueous electrolytes were described [27]. One of the first works related to PECs was carried out by Michaels A. and Miekka R. [28] in 1961 studying the formation and properties of the stoichiometric complexes of synthetic PEs− poly(4−vinylbenzyltrimethylammonium chloride) associated with sodium polystyrene sulfonate. PECs based on chitosan and CMS are of great importance in the development of new-generation environmentally friendly materials with low toxicity, high biocompatibility, and biodegradability [29,30]. Their formation occurs mainly due to electrostatic interactions between the positively charged amino groups of chitosan and negatively charged carboxymethyl groups of CMS. The advantages of the complexes are due to their improved stability, mechanical strength, and film formation properties compared to individual polymers. Also, such complexes can form gels or microcapsules, which open up opportunities for their application in biomedicine, food preservation, chemical detectors, membrane technology, fuel cells, and pharmaceuticals as effective carriers for drug delivery [30]. 

Some works have prepared and studied the use of PECs in drug delivery applications. For example, Quadrado F. and Fajardo A. [31] synthesized microparticles based on rice CMS with a degree of substitution of 0.5 and chitosan at room temperature, with a ratio of 3:4 for chitosan and CMS, respectively. These microparticles presented better chemical and thermal properties than the microparticles prepared by conventional ionotropic crosslinking. In addition, microparticles based on PEC showed more efficiency for drug encapsulation, leading to their use as vehicles for drug delivery systems. In another work, Assad E. et al. [32] prepared and characterized PECs of CMS and chitosan at a 1:1 ratio with a final pH of 5, and used them for drug delivery. The results of fluid diffusion and swelling in simulated gastric fluid or simulated intestinal fluid solutions were lower with PEC compounds in comparison with CMS:chitosan powder mixture. This allows the use of PECs as a drug carrier since it can prolong drug release time. In other research [33], polyelectrolyte complexation and ionotropic gelation methods were compared for obtaining hydrogels based on CMS and Chitosan. The hydrogels were prepared by mixing polyelectrolytes with a ratio of 16:1 for chitosan:CMS, respectively. Then, the hydrogel was kept for 24 h at room temperature until the equilibrium. Wang Y. et al. [34] demonstrated that the tablets made of PEC chitosan:CMS had lower swelling in acid media, and slower water uptake in comparation with the tablets prepared from independent polymers. In the study [35], devoted to the creation of PEC nanoparticles based on chitosan and carboxymethyl starch, their ability to package aminosalicylic acid (5-ASA) and release this substance in vitro was studied. The obtained chitosan:CMS nanoparticles containing 5-ASA had a high loading rate and 5-ASA could be released under controlled conditions, further proving the great potential of chitosan:CMS nanoparticles in targeted drug delivery, especially for the treatment of colorectal diseases. Also, in the work [36], polyelectrolyte complexes of chitosan with carboxymethyl starch were investigated and used as monolithic matrices with acetaminophen as excipients for directly pressed tablets. The mixture also included 5% bromocresol green as pH indicator to study drug behavior in gastric acid. It was shown that after the interaction of the tablets with simulated gastric fluid, the integrity of the tablets was maintained, thus achieving higher drug loading, providing useful properties for drug delivery. This study confirmed that chitosan-based polyelectrolyte complexes can be effectively utilized in biopharmaceuticals, therapeutic enzymes, and for probiotic delivery to the colon. 

As the literature shows, most of the studies are focused on the study of the application of PECs based on chitosan:CMS in drug delivery [31,32,33,34,35,36]. Perhaps there are not enough studies focused on the processes of the formation of PECs based on chitosan and CMS, and the influence of the molecular weight of CMS, DS, and biopolymer ratio has been poorly studied. Studies of the PEC formation process are important to know the optimal conditions such as pH, ratio, and polymer concentration to obtain PECs. For this reason, the main aim of the present research was to obtain a polyelectrolyte complex based on chitosan and carboxymethyl starch by the titration method depending on the type of carboxymethyl starch (CMS) and its ratio and study the process of formation. For this purpose, carboxymethyl starches were synthesized by the two-step reaction process of carboxymethylation with a constant amount of NaOH and different concentrations of monochloroacetic acid to obtain products with different degrees of substitution. The obtention of the PECs starch was carried on by mixing a diluted solution of CMS and chitosan and adjusting the pH until the obtention of the maximum formation of the (bio)PEC dispersions. The obtained (bio)PEC dispersions were characterized by dynamic light scattering and turbidimetry methods. The influence of the ratio of biopolymers, molecular weight, and degree of substitution were studied.

## 2. Materials and Methods

### 2.1. Reagents and Chemicals

Cassava starch (CS) (Thai Food King, Samutprakarn, Thailand) was used for the obtention of carboxymethyl starch (CMS), and chitosan (Chit) with low molecular weight (Bioprogress, Biokombinata, Russia) was used for the obtention of polyelectrolyte complex. Monochloroacetic acid (MCA) (NevaReactiv, Saint-Petersburg, Russia) was used for the carboxymethylation of starch. Distilled water was used as a solvent. Solutions of 0.1 M NaOH and HCl 0.1 M were used to set the pH.

### 2.2. Attenuated Total Reflectance Fourier-Transform Infrared Spectroscopy Analysis

The chemical composition of the used chitosan, cassava starch, CMS products, and dried (bio)PEC dispersions of Chit:CMS were analyzed by Fourier-transform infrared spectroscopy (FTIR) using an FTIR spectrometer Tensor 37 (Bunker, Karlsruhe, Germany) equipped with the attenuated total reflection (ATR) unit MIRacle (Pike, Madison, WI, USA) with a crystal of ZnSe. The ATR-FTIR spectra of the samples were obtained with a wavenumber range of 4000–500 cm^−1^ with an increment of 2 cm^−1^ as averages over 32 measurements.

The degree of acetylation (*DA*) of the used chitosan was calculated from the ATR-FTIR spectrum of chitosan powder using Equation (1) [37]

(1) $$DA=\frac{\frac{{A\;}_{1320}}{{A\;}_{1420}}-0.3822}{0.03133},$$

where *A*_1320_ and *A*_1420_ are the normalized absorbance at wavenumbers 1320 and 1420 cm^−1^, respectively. The degree of deacetylation (*DD*) was calculated by the difference in *DA* (Equation (2))

*DD* = 100% − *DA*. (2)

### 2.3. Determination of Acid Dissociation Constant of Chitosan

The acid dissociation constant *pK_a_* was determined by potentiometric titration using a pH meter S213-kit (Mettler Toledo, Giesen, Germany) in the range from 2 to 12. For this, a solution of 0.1 g of chitosan was prepared in 50 mL of 0.1 N HCl solution at 40 °C and mixed for 1 h. After this, the solution was titrated by separating 0.1 M NaOH solution until pH = 12. The pH and *pK_a_* were calculated using the modified Henderson–Hasselbalch Equations (3) and (4) [38]

(3) $$pH={pK}_{1/2}-p\;ln\left(\frac{1-\alpha }{\alpha }\right),$$

(4) $$pK_{a}={pK}_{1/2}-(p-1)\;ln\left(\frac{1-\alpha }{\alpha }\right),$$

where *p* is an empirical parameter related to the free energy of change during titration and α = 0.5 is the degree of dissociation (based on the volume of solution used in the titration). In addition, *pK*0 is the protonation constant at α→0 [38].

### 2.4. Carboxymethylation of Cassava Starch

**Figure 1:**
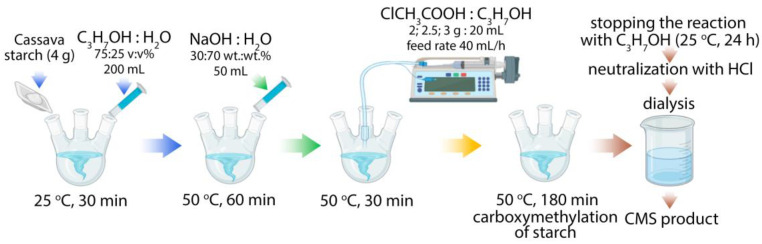
Scheme for synthesizing carboxymethyl starch products.

For the obtention of CMS, 4 g of cassava starch were dissolved in isopropanol water solution (75 *v*/*v*%) in a 250 mL three-necked flask and mixed by mechanical stirring under room temperature for 30 min. Then, 50 mL of NaOH (30 wt.%) solution was added at 50 °C for 60 min. Different amounts of 2, 2.5, and 3 g of monochloroacetic acid (MCA) were dissolved in 20 mL of isopropanol at room temperature for 30 min in order to synthesize three types of CMS. Then, 20 mL of the solution of MCA was gradually introduced into the reaction mixture at a feed rate of 40 mL/h using a syringe pump Sino MDT, SN-506 (Shenzhen, China) for 30 min. After that, the reaction progressed to 3 h at 50 °C. Finally, 200 mL of isopropanol was added to stop the reaction, and the product was sedimented for 24 h at room temperature. For the purification, the supernatants were separated and collated followed by removal and neutralization with HCl solution. After that, dialysis was carried out for 48 h with distilled water using an M-Cel dialysis bag with a pore diameter of 14 kDa (Rosmedbio, Saint-Petersburg, Russia) to remove salt from the products. Finally, the products were dried and named CMS 1, CMS 2, and CMS 3 in accordance with the amount of MCA used for their production. A detailed scheme for synthesizing the CMS products was drawn using the BioRender^®^ platform and presented in Figure 1.

#### 2.4.1. Establishing the Degree of Substitution of Carboxymethyl Starch

Direct titration was used for the determination of the degree of substitution (*DS*) of the obtained CMS products. For this purpose, 1 g of CMS for each type of product was dispersed in 50 mL of NaCl solution (2 wt.%), and then titrated with a solution of NaOH (1 M) until pH = 12 [39]. The *DS* values were calculated by Equation (5)

(5) $$DS=\frac{162\times {\;\eta }_{COOH}}{{m_{ds}-58\times \eta }_{COOH}},$$

where 162 is the molar mass of anhydroglucose unit (AGU), g/mol; *η_COOH_* amount of –COOH, mol, calculated from the equivalent volume of the known molarity solution of NaOH; 58 is the net increase in the mass of an AGU for each carboxymethyl group substituted, g/mol; and *m_ds_* is the dry mass of sample, g.

#### 2.4.2. Determination of Molecular Weight of Polymers

To establish the molecular weight of polymers, the method of capillary viscometry of dilute polymer solutions was used followed by the calculation of the viscosity-average molecular weight *M_η_* using the Mark–Kuhn–Houwink Equation (6) [40]

(6) $$\left[\eta \right]=K\times M_{\eta }^{\alpha },$$

where [*η*] is the intrinsic viscosity; *K* and α are constants that characterize the length and shape of the macromolecule, respectively. To determine the values of the intrinsic viscosity, dilute chitosan solutions in 0.33 M acetic acid/0.3 M NaCl mixture [41] and CMS solutions in 0.01 M NaCl with a concentration of 0.1–0.5 g/dL with an increment of 0.1 g/dL were prepared. Then, the flow time of the solutions and solvent through a glass thermostatic capillary with a diameter of 0.92 mm at a constant temperature of 25 °C was determined. Next, the values of specific and reduced viscosity were calculated. The values of [*η*] for chitosan and all the CMS products were determined using the Huggins approach, Equation (7), described in detail in the work [40]

(7) $$\eta_{r}=\left[\eta \right]+K_{h}\times {\left[\eta \right]}^{2}\times C,$$

where $\eta_{r}$ is the reduced viscosity and *K_h_* is the Huggins constant. Finally, the *M_η_* values were calculated according to Equation (6). For the used chitosan, the values of *K* and α constants were calculated by Equations (8) and (9) [41]

(8) $$Exp\;\alpha =\frac{0.6202\cdot x+0.699\cdot x}{0.4806+x},$$

(9) $$logK\cdot 10^{-5}=-5.7676\cdot Exp\;a+5.9232,$$

where *x* = *DA*/*pH·µ* and *µ* is the ionic strength of the solvent. For the CMS products, the values of *K* and α constants were selected based on the data from work [31] and were *K* = 2.28 mL/g and α = 0.277.

Also, using the values of [*η*] and *M_η_*, the apparent radius of gyration (*R*_g_^2^)^1/2^ of chitosan and CMS macromolecules was calculated using Equation (10) [42]

(10) $${\left(R_{g}^{2}\right)}^{1/2}=\sqrt[{3}]{\frac{3}{10\pi }\cdot \frac{\left[\eta \right]\cdot M_{\eta }}{N_{a}}\;},$$

where *N_a_* = 6.22 × 10^23^ is the Avogadro number.

### 2.5. Obtention of the Chitosan:Carboxymethyl Starch (Bio)Polyelectrolyte Complex Dispersions 

First, individual solutions of chitosan and CMS with a concentration of 0.25 wt.% were prepared by mixing in distilled water at 25 °C and 40 °C, respectively. The temperature with an accuracy of ±0.2 °C was controlled with a thermostatic thermometer ETS–D6 (IKA–Werke, Staufen, Germany). After mixing, the pH of the CMS solution was measured and readjusted until pH = 7 with a solution of 0.1 M HCl. The (bio)polyelectrolyte complex ((bio)PEC) dispersions between chitosan and all the CMS products were obtained by the mixing of individual solutions of polymers at different volume ratios of Chit:CMS: 1:1, 1:2, 1:3, 1:4, 1:5, and 1:6.

### 2.6. Characterization of the Chitosan:Carboxymethyl Starch (Bio)Polyelectrolyte Complex Dispersions

For the study of the pH influence in the formation of the Chit:CMS (bio)PEC dispersions, the change in the absorbance of all the individual solutions was analyzed. For this, each dispersion was titrated in the pH range from 2 to 12 by adding an aliquot of 0.1 M NaOH and 0.1 M HCl solutions, respectively. After each aliquot addition, the dispersion was mixed for 1 min, and the absorbance was measured with a spectrophotometer (Unico, Dayton, NJ, USA) at a constant wavelength of λ = 364 nm [43,44] and temperature of 25 °C. For the study of the particle size distribution of the initial chitosan, cassava starch, CMS products solutions, and Chit:CMS dispersions, the dynamic light scattering (DLS) method was used. In this connection, the refractive index *n* was determined with a refractometer IRF-454B2 M KOMZ (Kazan, Russia) for all the solutions. Using the values of *n*, the obtained solutions were diluted to 1:2 and measured using the particle size analyzer LB 550, Horiba (Edison New Jersey, NJ, USA) at 25 °C five times for each sample of solution. The final particle diameter distributions for the Chit:CMS (bio)PEC dispersions were obtained by averaging over the five measurements.

## 3. Results and Discussion

### 3.1. Physical and Chemical Characteristics and Structure of the Carboxymethyl Cassava Starch and Chitosan Solutions

**Figure 2:**
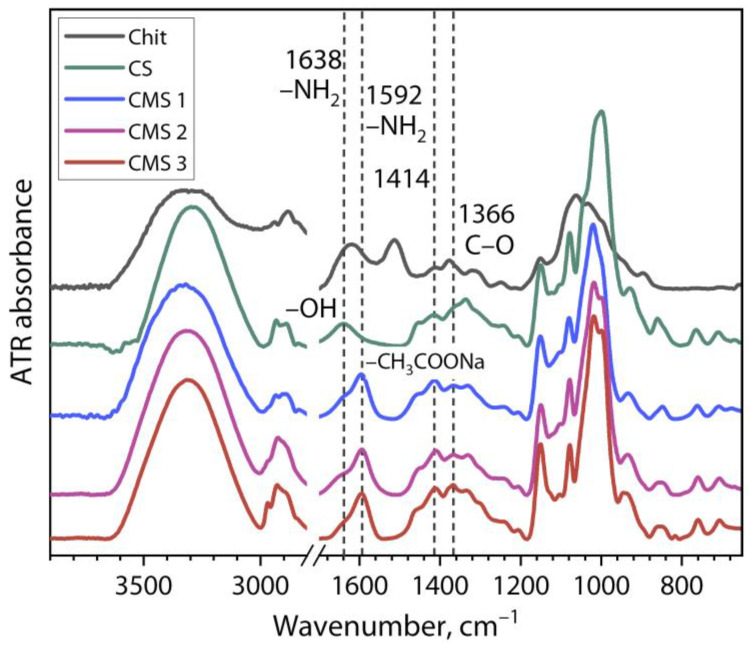
ATR-FTIR spectra of used chitosan, cassava starch, and obtained carboxymethyl starches.

Figure 2 shows the ATR-FTIR spectra of the initial chitosan and cassava starch powder samples and the obtained CMS powder samples. 

The presented spectra of polysaccharides show a region at 3700–3000 cm^−1^ that represents the stretching of hydroxyl groups. Another region at 3000–2800 cm^−1^ corresponds to C–H stretching. For the Chit, the spectrum is observed as a broad band with a center at 3349 cm^−1^ associated with –NH and –OH groups’ stretching vibration. The C–H stretching vibration of CH_2_ and CH_3_ is represented by the bands in the region of 2928–2856 cm^−1^. The bands at 1622 cm^−1^ and 1516 cm^−1^ correspond to the C=O stretching vibrating and N–H deformation, receptively. Peaks associated with the amino group in chitosan around 1592 cm^−1^ and *N*-acetylated chitin at 1638 cm^−1^ can be observed [31,43]. For the cassava starch, the band at 1638 cm^−1^ corresponds to the scissoring of two –OH bonds of absorbed water molecules [7,11]. In the case of the CMS samples, the substitution with the –CH_3_COONa salt radicals on the cassava starch is represented by the bands at 1592 cm^−1^ and 1414 cm^−1^ [12]. Also, the new bands between 1400 cm^−1^ and 1300 cm^−1^ correspond to the anti-symmetrical and symmetrical vibrations of the –COO^−^ structure due to two C–O bonds that would receive resonance effects as a result of the ionization [11]. 

Using Equations (1) and (2) based on the ATR-FTIR spectrum, the value of the degree of deacetylation *DD* of the used chitosan was established. Also, using Equation (5), the values of the degree of substitution *DS* of the obtained CMS products were calculated. The specified values were collected and presented in Table 1.

From Table 1, the value of the *DD* of the chitosan is greater than the *DS* values of the CMS products. This difference indicates that the chitosan, in proportion, has more available side amino groups for ionization than the CMS. Therefore, it can be seen that an increase in the amount of MCA in the carboxymethylation process leads to an increase in the *DS*. We believe that this increase is due to the fact that during CMS synthesis, there is a greater amount of carboxymethyl groups that are substituted to the starch chain, generating new active sites. In addition, the acid dissociation calculated constants of the chitosan from Equations (3) and (4) are *pK_a_* = 6.41 and *pK*_0_ = 6.34. These values are consistent with the literature data when *pK_a_* varies in the range of 6.20–6.50 and represents, for *pK_a_*, the pH at which half of the amino groups in chitosan are protonated, while the *pK*_0_ is the pH at which the net charge density of the polymer is altered in complex formation [38,44].

**Figure 3:**
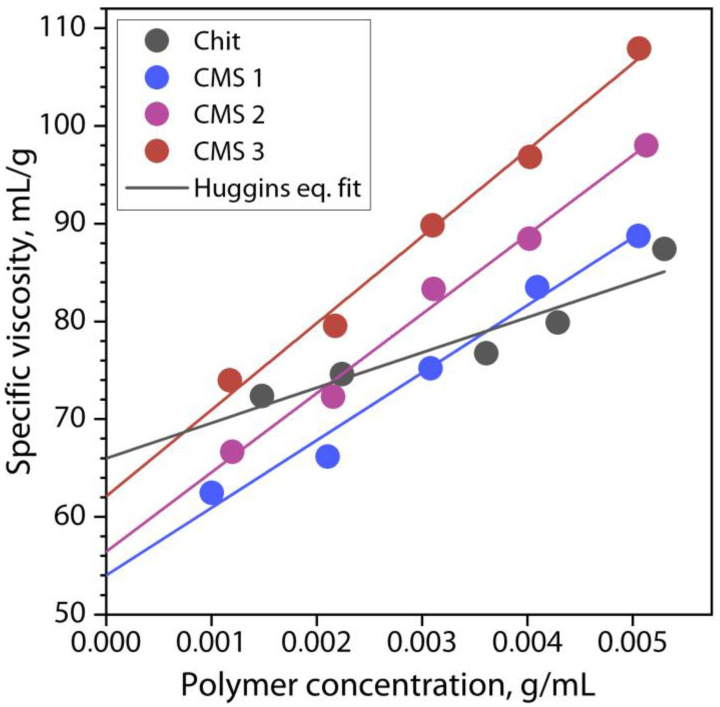
Reduced viscosity of chitosan and carboxymethyl starch solutions versus concentration.

Figure 3 shows the dependence of the reduced viscosity of the solutions of chitosan and CMS products on their concentration in salt buffer medium.

Dependence demonstrates a linear increase in the reduced viscosity of the solution with increasing polymer concentration, which indicates a high affinity of the polymers and the solvent system. Also, the increasing nature of the dependence indicates a suppressed effect of the electrolytic swelling of polyelectrolyte macromolecules in the presence of a salt buffer and allows us to establish the unperturbed size of uncharged macromolecules and their molecular weight. The obtained dependences are well described by the Huggins equation with *R*^2^ values greater than 0.9 (Table 1), which allowed us to establish the values of the Huggins constant *K_h_* and intrinsic viscosity [*η*], as well as calculate the viscosity-average molecular weight of the polymer *M_η_* and mean-square radius of gyration of macromolecule (*R_g_*^2^)^1/2^. The parameter values are presented in Table 1. It is clear from the table that the *K_h_* value for the chitosan and CMS samples is less than 0.5, which is usually typical for good solvents [45]. It is also evident that the molecular weight of the CMS products is several times higher than the value for the low molecular weight water-soluble chitosan used. At the same time, with an increase in the amount of MCA used in the carboxymethylation of cassava starch, the formation of a CMS product with an increase in *DS* and *M_η_* occurs. These results can be explained by the presence of negatively charged carboxymethyl groups in CMS, the fraction of which increases with an increase in the degree of substitution. In addition, the lowest value of (*R_g_*^2^)^1/2^ for chitosan molecules in the buffer solution is also characterized due to the compact chain and the small molecular weight of chitosan in comparison with CMS.

**Figure 4:**
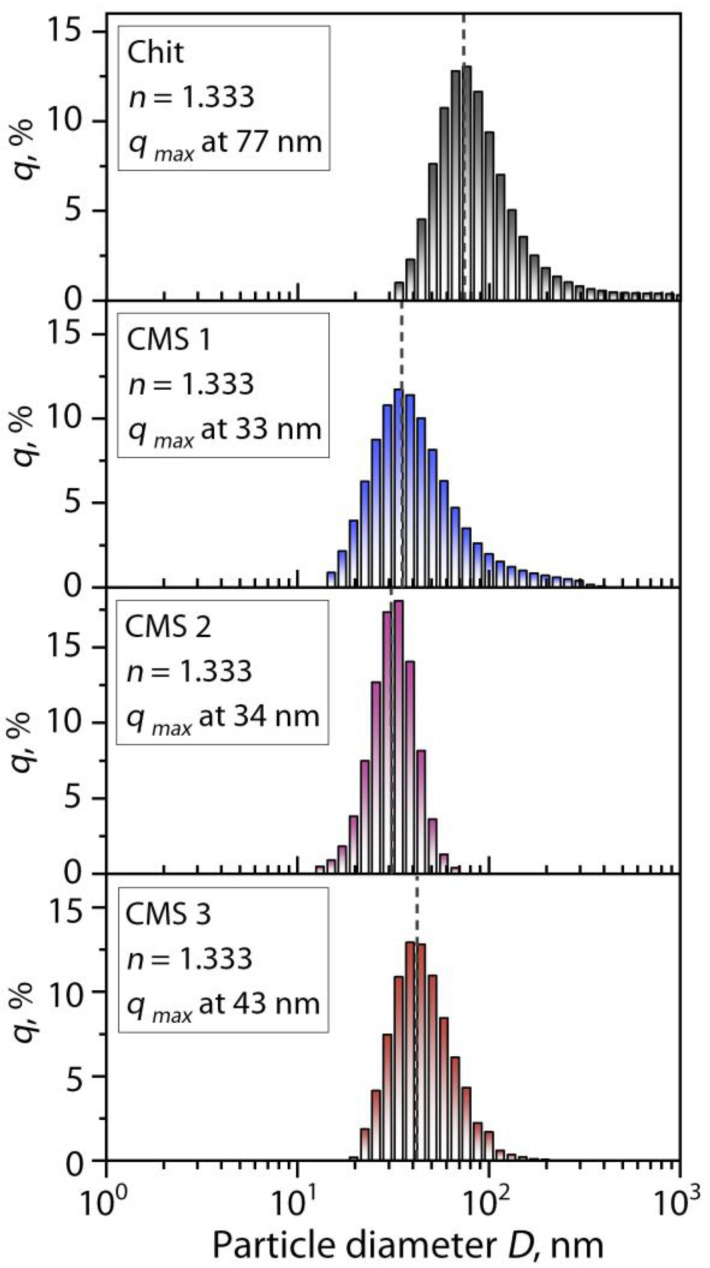
Particle diameter distributions of chitosan and carboxymethyl starch solutions in distilled water at 0.25 wt.%.

The dynamic particle diameter distribution for the individual solutions of chitosan and CMS products in distilled water is shown in Figure 4. The refractive index *n* values of the solutions are presented in the boxes. The particle size distribution (frequency distribution %) vector is represented by *q*. 

From Figure 4 it can be seen that the used chitosan and CMS product solutions are characterized by the monomodal distribution of particle diameters with different widths. It is noticeable that a small number of submicron-sized particles are also observed in the chitosan solution, which significantly exceeds the size of chitosan macromolecules as well as a large average particle diameter *D*_avg_ (average diameter of particles at *q*_max_) compared to its low molecular weight (Table 1). This trend is explained by the aggregation of chitosan macromolecules in a water solution. The main causes of chitosan aggregation in aqueous solutions can be both the association of *N*-acetyl-*D*-glucosamine units due to hydrophobic interactions and the hydrophilic interaction of *D*-glucosamine units with the formation of hydrogen bonds [46]. Aggregation occurs when the hydrophobic association of macromolecules overcomes the electrostatic repulsion of charged groups. It is evident that the *D*_avg_ values and the width of the distributions for the CMS samples are significantly smaller. The reason for this is probably the increased solubility of CMS macromolecules in water due to the presence of side carboxymethyl groups [47,48,49]. This shows that CMS macromolecules in water are not prone to aggregation, like chitosan molecules, and can be separate units. Therefore, for CMS solutions, it becomes possible to determine the hydrodynamic radius of macromolecule *R_h_* = *D*_avg_/2. The calculated values of *R_h_* are presented in Table 1. The values of *R_h_* demonstrate proportionality with the values of *DS*, *M_η_*, and (*R_g_*^2^)^1/2^ and increase with an increase in the mass of MCA used in the carboxymethylation of cassava starch. Another work [50] also showed that increasing the degree of substitution of CMS from potato starch from 0.40 to 0.81 causes an increase in [*η*], *R_g_*, and *R_h_* due to the presence of carboxymethyl groups.

### 3.2. Effect of the Biopolymer Ratio Chit:CMS v/v% in the Formation of PEC

**Figure 5:**
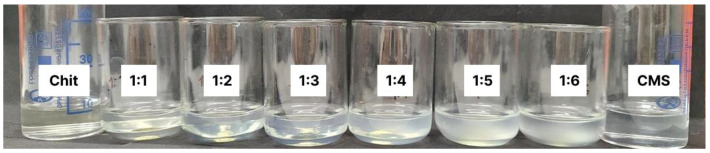
Visual state of chitosan, CMS, and their mixture solutions in distilled water at different Chit:CMS ratios.

The appearance of individual solutions of chitosan and CMS, as well as their mixtures at different Chit:CMS ratios, are shown in Figure 5.

The figure shows that individual polymer solutions are transparent, while their mixtures exhibit significant light scattering. It can be seen how an increase in the fraction of CMS in the mixtures leads to an increase in the turbidity of the solutions. This effect qualitatively reflects the process of polyelectrolyte interaction and may be associated with the formation and increase in the size of (bio)PEC structures between polyelectrolytes. 

This interaction can be expressed using the ratio of ionized groups z, which depends on the content of ionized functional side groups in the polymer. At *z* = 1, the PEC is stoichiometric and a balance of charges in the system is observed (the most effective polyelectrolyte interaction). On the contrary, under conditions *z* < 1 and *z* > 1, non-stoichiometric interactions between polyelectrolytes are presented, and there is an excess of positive and negative charge, respectively. The ratio of *z* was determined using Equation (11) [51]

(11) $$z=\frac{\left[{COO}^{-}\right]}{\left[{NH}_{3}^{+}\right]},$$

where [*COO*^−^] and [*NH*_3_^+^] are a molar concentration of charged units. The values of *z* were calculated taking into consideration the *DD* of chitosan and *DS* values of CMS. The results of the calculation of the *z* values for various compositions of (bio)PEC are presented in Table 2.

It can be seen from Table 2 that for all the mixtures of Chit:CMS 1 with the lowest *DS*, the formation of a (bio)PEC with an excess of positive charge *z* < 1 is observed (non-stoichiometric PEC). On the contrary, for mixtures with CMS 2 and CMS 3 with higher *DS* values, there is a transition from an excess of positive to an excess of negative charge with an increase in the proportion of CMS in the mixture observed (*z* < 1 and *z* > 1). A similar charge transition was also observed in work [52] for oligochitosan (*M_η_* = 5 kDa) and CMS PEC based on zeta potential measurement results. For example, it has been shown that when the ratio of polymers changes from 1:4 to 3:7, the zeta potential increases from −11.2 to 4.8 mV, correspondingly. This is correlated with the results of calculating the ratio of ionized groups in PEC.

The formation of non-stoichiometric polyelectrolyte complexes has been reported by some works [53,54,55,56,57,58,59]. Typically, these PECs are prepared by mixing polyelectrolytes of significantly different molecular weights in non-stoichiometric proportions. The obtained particles consist of a long host macromolecule complexed with shorter molecules of a guest polyelectrolyte with an opposite charge [60]. Drogoz et al. [56] studied the formation of polyelectrolyte complexes of polysaccharides using chitosan and dextran sulfate. The obtained dispersions had a negative or positive charge depending on the excess polyelectrolyte, and it was shown that to maintain the stability of the dispersions, the z value should be less than 0.6. In addition, the non-stochiometric PEC of modified chitosan with polystyrenesulfonate was investigated by Izumrudo A. [57]. In this work, the critical ratio (z_cr_ = 0.3) was determined by titration; at this point, a slight turbidity was observed and it was also shown that the addition of one of the polyelectrolytes allows the formation and accumulation of insoluble PEC. These results confirm the formation of the insoluble complexes obtained in our work when z ≠ 1.

**Figure 6:**
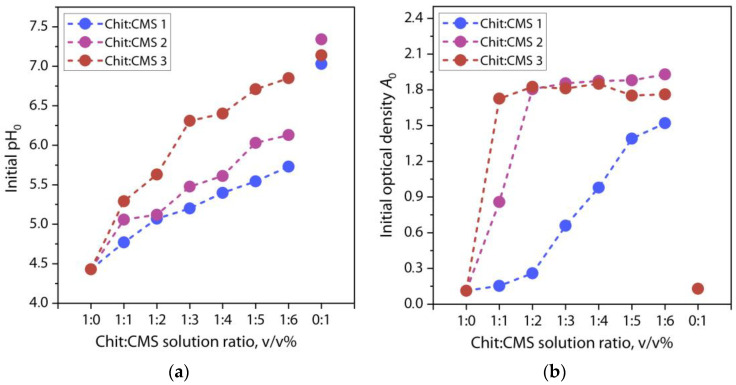
Dependences of (**a**) *pH*_0_ and (**b**) optical density of solutions on the Chit:CMS ratio.

Optical density was measured to study the effect of the CMS type and the ratio of ionized groups on the formation of (bio)PEC dispersions. The relationship between the initial pH value of the Chit:CMS mixtures after preparation *pH*_0_ and their optical density versus Chit:CMS ratio are shown in Figure 6.

For a better understanding of the influence of the biopolymer ratio in solution, it is important to analyze the change in the initial *pH*_0_. Figure 6a shows that the pH values of the initial chitosan and CMS solutions are approximately 4.3 and 7.17, correspondingly. Therefore, with the increase in the fraction of the CMS solution, the pH of the mixture increases. However, it is noteworthy that the character of the change in pH values depends on the *DS* value of CMS. For example, for mixtures with CMS 3 with a higher *DS* the pH values increase more intensively compared to mixtures with CMS 1. This happens because with an increase in *DS* of CMS, many negatively charged carboxymethyl ions become available to positively charged amino groups of chitosan which reduces the amount of H^+^ ions producing an increase in pH [33,61,62]. Figure 6b shows that mixing individual solutions of chitosan and CMS, which have no optical density, results in a sharp increase in optical density, which is confirmed by the visual observations of the mixtures (Figure 5). Moreover, when using CMS with a high *DS* value, the optical density of the mixture solutions increases sharply to plateau values, for example, compared to the mixture with CMS 1. This observation confirms the increase in *DS* for CMS products with an increase in the proportion of MCA during their production, and also indicates a more efficient formation of (bio)PEC structures when using CMS 2 and 3 [2,63,64].

### 3.3. Characterization of (Bio)Polyelectrolyte Complex

**Figure 7:**
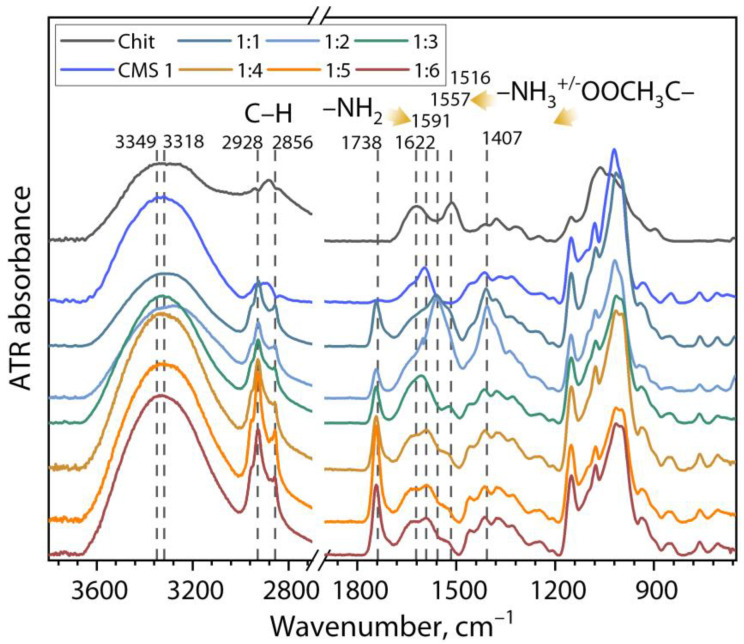
ATR-FTIR spectra of dried (bio)PEC dispersion samples at different ratios of Chit:CMS 1.

Figure 7 shows the ATR-FTIR spectra of the chitosan, CMS 1, and dried (bio)PEC dispersion samples at different ratios of Chit:CMS 1.

On the spectrum of Chit:CMS 1 (bio)PEC samples, new absorption bands and more intensive peaks are shown in comparison with the Chit and CMS 1 spectra. One significant change is the narrowing of the bands with a center around 3349–3318 cm^−1^. This can be associated with the formation of hydrogen bonds between the Chit and CMS macromolecules, as well as with the electrostatic interaction between the amino groups of chitosan and the carboxymethyl groups of CMS in the formation of (bio)PECs [43,65,66]. In addition, the absorption peaks at 2928 and 2865 cm^−1^ became sharper. This could be attributed to the C–H stretching vibration becoming more intensive, likely due to the CH_2_ groups of CMS [31,67,68]. Another change observed in the samples with a high fraction of CMS (1:4, 1:5, and 1:6) is the appearance of shoulder bands in the region of 1635–1591 cm^−1^ related to the stretching vibration of the C=O from CMS. In addition, the bands at 1557 cm^−1^ are likely due to –NH_3_^+^ groups in chitosan. Moreover, the interaction between chitosan and CMS may happen by hydrogen bonds and ionic interactions to form (bio)PEC which is shown in the bands at around 1738 and 1557 cm^−1^ [31,32].

### 3.4. Effect of the pH on the (Bio)Polyelectrolyte Complex

**Figure 8:**
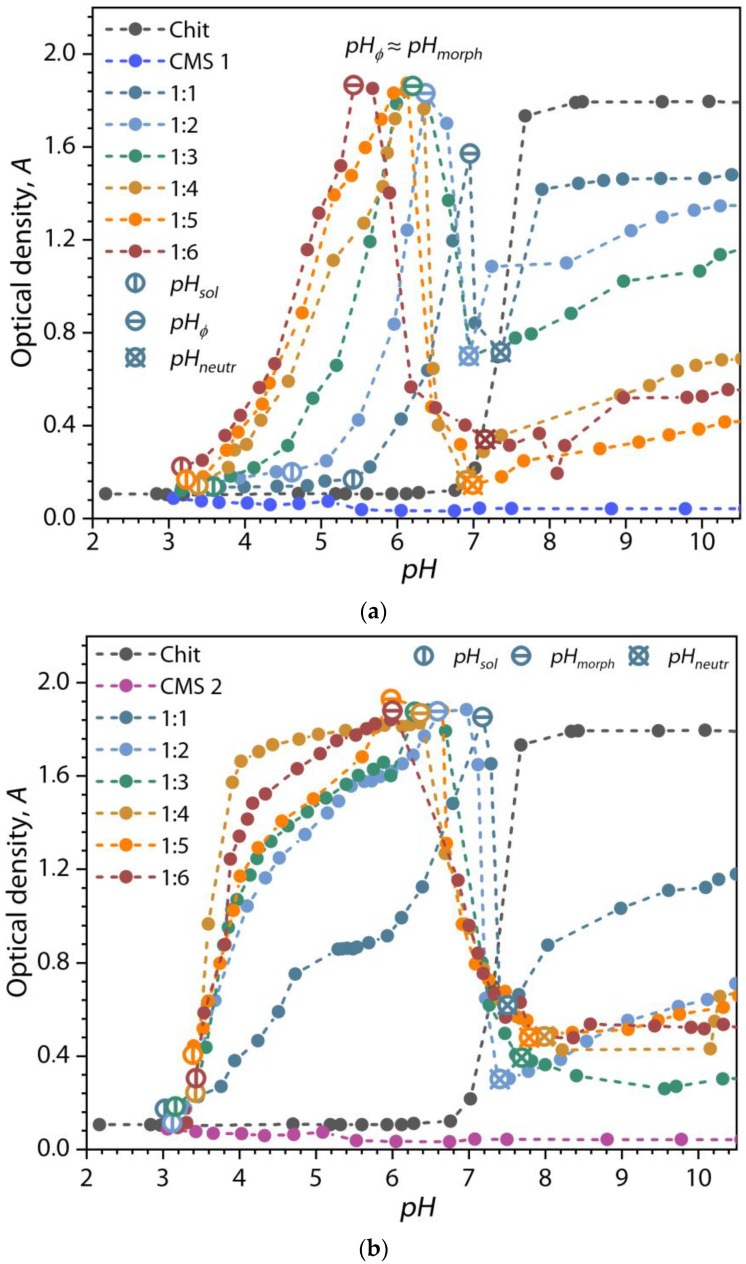

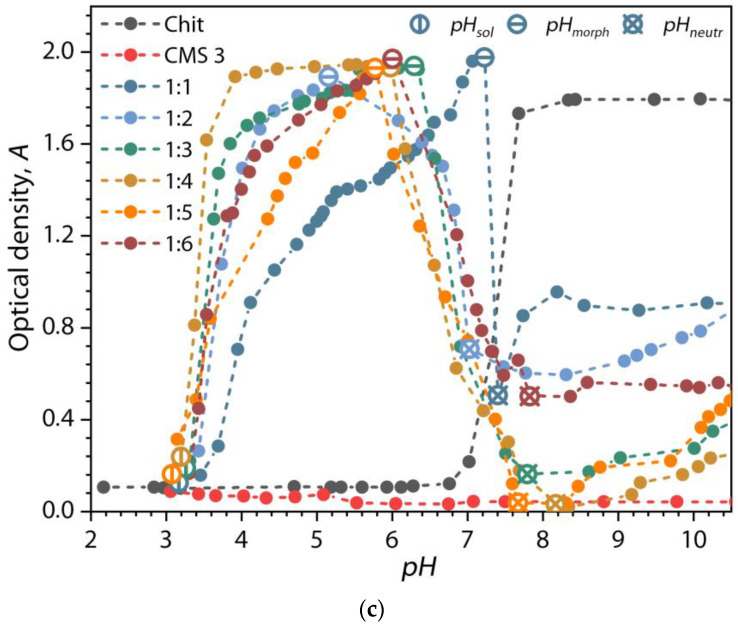
Effect of the pH on the optical density with (**a**) CMS 1, (**b**) CMS 2, and (**c**) CMS 3.

The turbidimetric titration of the mixtures of chitosan and CMS solutions was carried out to clarify the ranges of formation of the (bio)PEC structures and their stability. The influence of pH on the optical density of chitosan, CMS, and Chit:CMS mixtures with different CMS products is illustrated in Figure 8.

At first, for the chitosan solution, it can be observed that there are no changes in the optical density (*A*) up to a pH of seven (more than *pK*_0_ = 6.34). Above this point, there is a sharp increase in the values of *A*, which indicates the formation of a microphase dispersion of chitosan and causes its degree of protonation to be reduced [69,70]. On the other hand, for the CMS solutions, the *A* has not significantly changed because of their chemical structure. It is known that at the degree of substitution (carboxymethylation) of starch *DS* ≥ 0.2, CMS dissolves well in water [71]. Moreover, from the dependencies, it is clear that even under strongly acidic conditions, CMS remains in a single-phase solution and does not precipitate into a microphase or sediment. In the case of mixtures of chitosan and CMS solutions, we observe an increase in the *A* values uncharacteristic for individual polymer solutions. This, as previously shown, is due to the formation and accumulation of (bio)PEC particles.

For the Chit:CMS (bio)PEC dispersions, four zones delimited by pH transition points can be identified according to the method described in works [51,72]. The first zone is delimited by the *pH* ≤ *pH_sol_*. In this zone, the *A* is constant and the solutions are almost transparent which is associated with the formation of unstable and soluble PEC structures. The second zone is *pH_ɸ_*–*pH_morph_*, which corresponds to the formation of insoluble PEC dispersion, where a strong increase in *A* value is seen compared to the individual polymer solutions. It is noteworthy that the position of the *pH_ɸ_* change depends on the *DS* of CMS. For example, for Chit:CMS 1 mixtures (Figure 8a), as the ratio increases from 1:1 to 1:6, the *pH_ɸ_* values decrease from 5.6 to 3.4, respectively. However, for mixtures with CMS 2 and CMS 3 the *pH_ɸ_* values are weakly dependent on the polymer ratio. We believe that this is due to an increase in the content of ionized carboxymethyl groups in CMS with an increase in *DS*, which leads to a decrease in pH values necessary for the effective formation of PEC particles. With a further increase in pH, we observe a maximum optical density, which corresponds to the value of *pH_morph_*. At this point, the stoichiometric complexation is achieved (n^+^ ≈ n^−^) and the polyelectrolyte interaction process occurs most efficiently.

It is important to note that in cases where both oppositely charged polyelectrolytes have a relatively high degree of substitution with charged functional groups, *pH_morph_* may not be a single point but a range of pH values (plateau of *A* values). In such cases, the dispersion of (bio)PEC particles may be effectively formed over a wide pH range. It can be seen from the figures that the *pH_morph_* range changes depending on the type of CMS. So, for example, for mixtures of Chit:CMS 1, *pH_morph_* is a single point, but for mixtures of Chit:CMS 2 and Chit:CMS 3, this is a range of 4.9–6.4 and 4.3–6.4, respectively. This phenomenon may be because at higher *DS* of CMS, more carboxymethyl groups are present for complexation with the amino groups of chitosan. As a result, this leads to an increase in the stoichiometry of the complex structures when the *z* value is close to one or higher. In this regard, for mixtures of Chit:CMS 1, the values of *z* increase slightly with increasing polymer ratio and the *pH_morph_* point decreases, while the area under the curve *A* (pH) increases strongly, which is associated with an increase in the concentration of ionized carboxymethyl groups in the solution despite the low value of *DS* of this CMS. At the same time, in mixtures with CMS 2 and 3 with higher values of *DS*, with an increase in the proportion of CMS in the solution, the value of *z* increases more intensively, which leads to an increase in the range of *pH_morph_* and a large area under the curve *A*(pH) even at low Chit:CMS ratios.

In the next zone, when *pH_morph_*–*pH_neutr_* the (bio)PEC structures start to collapse and degrade. As can be seen from Figure 8, the pH values are close to *pK*_0_ for chitosan, which links the effect of decreasing *A* for dispersions with the process of the deprotonation of chitosan amino groups under alkaline conditions, leading to the destruction of (bio)PEC structures. In this range, the dispersion contains soluble (bio)PEC and CMS particles, and insoluble chitosan particles [73,74,75]. For the last range at pH > *pK*_0_, the dispersion contains insoluble chitosan microphase, as mentioned above, and dissolved CMS macromolecules.

### 3.5. Structure of (Bio)PEC Dispersions Based on Chitosan and Carboxymethyl Starch

**Figure 9:**
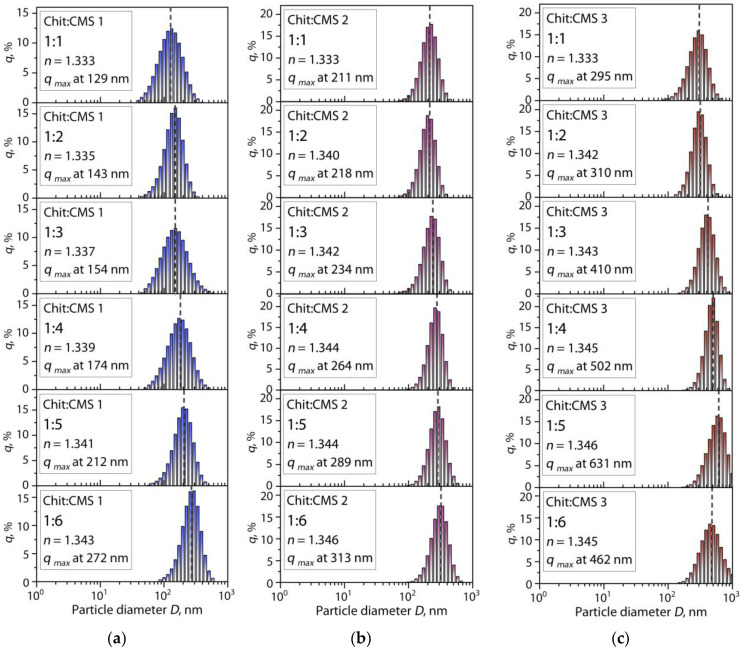
Particle diameter distributions for Chit:CMS (bio)PEC dispersions at *pH_morph_* with different ratios of chitosan and (**a**) CMS 1, (**b**) CMS 2, and (**c**) CMS 3.

The study of the effect of pH on Chit:CMS mixtures gives a qualitative idea of the formation of (bio)PEC dispersions and shows the pH range necessary to achieve stable dispersions. However, by means of this analysis, it is not possible to analyze the structure and particle size of the (bio)PEC particles. For this reason, the dynamic light scattering method was used in order to analyze the particle diameter distribution of the (bio)PEC dispersions at *pH_morph_* (Figure 9). The *n* values of the solutions are presented in the boxes.

From Figure 9, it can be seen that all of the ratios are characterized by monomodal distribution and this distribution reminds the result for the individual biopolymer solutions (Figure 4). In addition, it is observed that the average particle diameter *D*_avg_ of the (bio)PEC dispersions is bigger than the sum of it for the independent macromolecules of chitosan and CMS. This effect is probably due to the intermolecular interaction of the polymers following its complexation. The main observed effect is associated with an increase in the value of *D*_avg_ with an increase in the ratio of Chit:CMS in dispersions for all types of the obtained CMS. At the same time, it is evident that the relative width of all the distributions (within one order of diameter) is similar, which means the isotropy of the formed particles and the absence of their coagulation. The observed effects indicate that an increase in *D*_avg_ is mainly associated with an increase in *DS* and the proportion of CMS in the composition of the mixtures.

Some works [76,77,78,79,80] report the same form of distribution for PECs, attributing this to the neutralization of the charges of the polyelectrolytes, which decreases the repulsive forces and leads to an increase in particle size. For example, in the work [76], microgels from different substituted CMS showed a monomodal distribution of the particle diameter that became larger with the increase in *DS* because of the larger amounts of carboxymethyl groups. Li et al. [77] obtained complex nanogels from chitosan hydrochloride and carboxymethyl starch. It was noted that the increase in CMS content might induce an increase in particle size because of an increase in interaction between polymers. 

**Figure 10:**
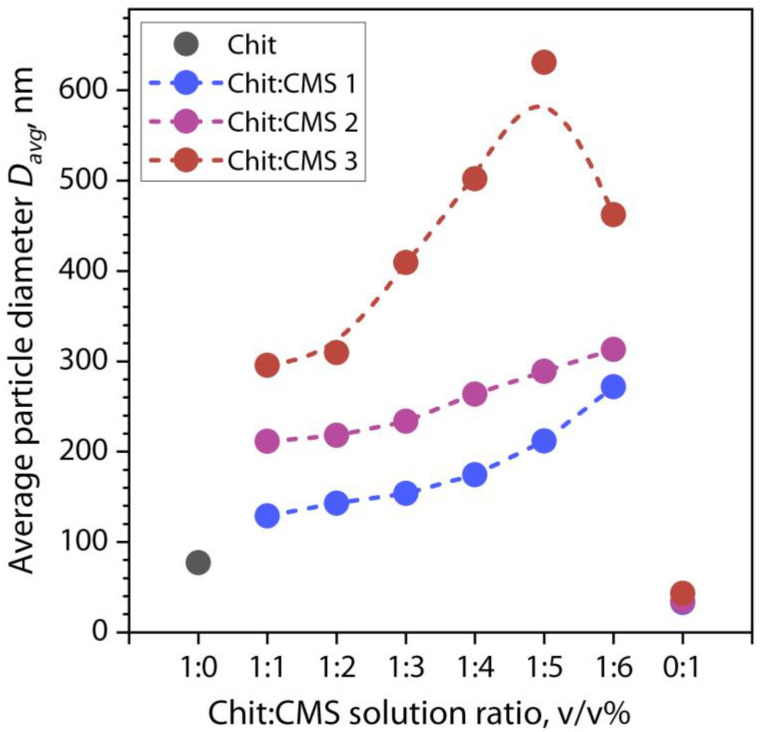
Effect of the Chit:CMS ratio and type of CMS at *pH_morph_* on the average diameter at qmax for the dispersions of (bio)PEC.

For a better understanding of the influence of the Chit:CMS ratio and the type of CMS on the particle diameter, Figure 10 shows the effect of the ratio at *pH_morph_* on the average diameter *q_max_* for the dispersion of (bio)PEC.

From Figure 10, it can be seen that the *D*_avg_ value increases with the fraction of CMS for mixtures of Chit:CMS with all types of CMS. In addition, with an increase in the *DS* and *M_η_* values for CMS, a general increase in *D*_avg_ is observed regardless of the proportion of CMS in the mixture. With an increase in the proportion of CMS in the Chit:CMS 1 and Chit:CMS 2 mixtures from 1:1 to 1:6, the *D*_avg_ value increases by 143 nm and 102 nm, respectively. Interestingly, in the Chit:CMS 3 mixture, this effect is more intense, and an increase in the proportion of CMS 3 in the mixture from 1:1 to 1:5 leads to an increase in *D*_avg_ by 335 nm. This is probably because this mixture is in the studied range of polymer ratios *z* ≥ 1 compared to the use of CMS 1 and 2 (Table 2). However, with an increase in the Chit:CMS 3 ratio from 1:5 to 1:6, there is a decrease in the *D*_avg_ value, which is explained by a strong excess of negative charge of carboxyl groups of CMS at *z* > 1 (Table 2). This leads to the fact that the formed negatively charged particles of primary PEC aggregate worse and do not form larger particles as in the case of *z* ≈ 1. A similar dependence of the decrease in the diameter of the PEC particles was also observed in a number of works [50,78,81,82,83]. 

Comparing our results with other studies carried out, for example, those by Saikia C. et al. [84] who obtained carboxymethyl starch-chitosan-coated iron oxide magnetic nanoparticles with a particle size distribution of 219–247 nm, it can be seen that our particles (CMS 2) can also be used in encapsulation applications for drug delivery. Furthermore, Wu D. and Delair T. [85] obtained electrolyte complexes based on chitosan/hyaluronan and studied the effect of factors such as pH and polymer ratio. It can be observed that, as in our work, an increase in the polymer ratio leads to an increase in the average particle size, but with a DA 16% chitosan, an excess in this ratio decreases the particle size due to the charge shift in the electrolytes. Moreover, in the work [86], the stability of the PECs based on chitosan:alginate with respect to pH changes was also evaluated. For these dispersions, the most significant changes were noted in the pH range 6.4 to 7, while at pH > *pKa* weak electrostatic interactions occur, causing aggregate formation. This is in agreement with our optical density results which show a reduction in its value at pH higher than *pKa*.

## 4. Conclusions

In the present work, biopolyelectrolyte complex (bio)PECs based on chitosan (Chit) and carboxymethyl starch (CMS) were obtained by mixing independent biopolymer solutions. The effect of the ratio of biopolymer and of ionized groups *z*, pH, and degree of substitution of CMS in the formation of the (bio)PECs were studied by the turbidimetry and dynamic light scattering (DLS) methods. For this purpose, three carboxymethyl starches were synthesized. The ATR-FTIR spectra of these products showed new bands at 1592 cm^−1^ and 1414 cm^−1^, representing the substitution with –CH_3_COONa salt radicals on the cassava starch. In addition, the values of *DS* were 0.16, 0.33, and 0.36 for CMS 1, CMS 2, and CMS 3, respectively. 

For the preparation of (bio)PECs, independent solutions of Chit and CMS with 0.25 wt% were mixed in ratios from 1:1 to 1:6 *v*/*v*%, with an increment of 1 in the amount of CMS. The formation of the (bio)PEC was confirmed by the increase in optical density after mixing the individual solutions of Chit and CMS. Furthermore, the increase in the fraction of CMS in the mixtures leads to an increase in the size of (bio)PEC structures between polyelectrolytes. Therefore, the use of CMS 2 and CMS 3 with a higher *DS* increases the efficient formation of (bio)PEC. In addition, in the ATR-FTIR spectra appeared new bands at around 1738 and 1557 cm^−1^, which confirmed the interaction between Chit and CMS.

The formation of (bio)PECs is divided into four zones delimited by pH transition points. The first zone corresponds to the formation of unstable and soluble PEC structures. The second zone corresponds to the formation of insoluble PEC dispersion, where *DS* of the CMS influences the *pH_ɸ_*. The final pH point of this second zone is the *pH_morph_*, the point where the stoichiometric complexation is achieved (*z* = 1) and the polyelectrolyte interaction process occurs most efficiently. This *pH_morph_* depends on the type of CMS. For Chit:CMS 1, *pH_morph_* is a single point, and for CMS 2 and CMS 3, this is a range of 4.9–6.4 and 4.3–6.4, respectively. The third zone corresponds to the collapse and degradation of (bio)PECs. And, the last zone is characterized by an insoluble chitosan dispersion with dissolved CMS. The results of the dynamic light scattering method have shown a monomodal distribution, similar to the initial biopolymers, for all ratios of polymers and for all types of CMS. Moreover, an increase in the ratio of Chit:CMS in dispersions for all types of the obtained CMS leads to an increase in the value of *D*_avg_. But, in the dispersion with the higher *DS* (CMS 3) at a value of *z* ≥ 2, this effect is annulated, and a slight decrease is appreciated due to the too-low density of ionic bonds between polyelectrolyte radicals.

## Figures and Tables

**Table 1:** Molecular characteristics of the used chitosan and CMS products.

Polymer	MCA, g	DS (or DD for Chit)	*K_h_*	*R* ^2^	[*η*], mL/g	*M_η_*, kDa	*(R_g_*^2^)^1/2^, nm	*R_h_*, nm
Chit	–	0.82	0.16	0.90	66.1	30	7	–
CMS 1	2.0	0.16	0.24	0.98	54.0	92	9	17
CMS 2	2.5	0.33	0.25	0.99	56.4	107	10	17
CMS 3	3.0	0.36	0.23	0.99	62.0	151	11	22

**Table 2:** The effect of the ratio of ionized groups on the composition of PEC with different types of CMS.

Chit:CMS	Ratio of Ionized Groups *z*		
	CMS 1	CMS 2	CMS 3
1:1	0.10	0.21	0.32
1:2	0.20	0.42	0.65
1:3	0.30	0.64	0.98
1:4	0.40	0.85	1.30
1:5	0.50	1.06	1.63
1:6	0.60	1.27	1.96

## Data Availability

Data are contained within the article.
